# Polyphenol-Containing Feed Additive Polygain™ Reduces Methane Production and Intensity from Grazing Dairy Cows Measured Using an Inverse-Dispersion Technique

**DOI:** 10.3390/ani15070926

**Published:** 2025-03-24

**Authors:** Mei Bai, Pragna Prathap, Muhammed Elayadeth-Meethal, Matthew Flavel, Richard Eckard, Frank R. Dunshea, Richard Osei-Amponsah, Mohammad Javed Ashar, Deli Chen, Surinder Chauhan

**Affiliations:** 1 School of Agriculture, Food and Ecosystem Sciences, Faculty of Science, The University of Melbourne, Parkville, VIC 3010, Australia; pragnaprathap@gmail.com (P.P.); muhammed.elayadethmeethal@unimelb.edu.au (M.E.-M.); rjeckard@unimelb.edu.au (R.E.); fdunshea@unimelb.edu.au (F.R.D.); rich12668@yahoo.co.uk (R.O.-A.); javed.mohammadjavedashar@student.unimelb.edu.au (M.J.A.); delichen@unimelb.edu.au (D.C.); ss.chauhan@unimelb.edu.au (S.C.); 2 The Product Makers (Australia) Pty Ltd., 50–60 Popes Rd., Keysborough, VIC 3173, Australia; mflavel@tpm.com.au; 3 Department of Microbiology, Anatomy, Physiology and Pharmacology, La Trobe University, Bundoora, VIC 3083, Australia; 4 School of Biology, Faculty of Biological Sciences, The University of Leeds, Leeds LS2 9JT, UK; 5 Department of Animal Science, School of Agriculture, College of Basic and Applied Sciences, University of Ghana, Legon, Accra P.O. Box LG 226, Ghana

**Keywords:** dairy cows, methanogenesis, inhibition, Polygain, IDM

## Abstract

This study, conducted on a commercial dairy farm using a robotic milking system in Victoria, Australia, examined the effects of Polygain™ (The Product Makers Australia), a polyphenol-rich sugarcane feed material (PRSFM), on CH_4_ emissions in grazing dairy cattle using an inverse-dispersion model (IDM) combined with open-path laser techniques. Thirty lactating Holstein Friesian cows (aged 2–5 years with an average body weight of 663 kg and average daily milk production of 28.9 kg) were divided into two dietary treatment groups of fifteen cows each. Before the measurement, the PRSFM (0.25%) was supplemented for 3 weeks as an adaptation period and mixed with pellet feed for the treatment group. Over the 2-week measurement period, CH_4_ production (MP) was 495 ± 12 and 377 ± 12 (mean ± standard error) g CH_4_/animal/day for the control and treatment groups, respectively. Methane intensity (MI) was 17.04 and 13.01 g CH_4_/animal/kg milk/day in the control and treatment groups, respectively. On average, Polygain supplementation reduced MP and MI by 24%. This potential CH_4_ reduction extrapolated across Australia contributes to a 2.63% reduction in national agricultural emissions. This study underscores the potential of Polygain for CH_4_ mitigation in dairy cattle.

## Simple Summary

Enhanced atmospheric methane (CH_4_) emissions contribute to environmental pollution, accelerate global climate change, and increase energy loss in feed. The Australian dairy industry accounts for 12.5% of greenhouse gas (GHG) emissions in the agriculture sector, and the Australian government aims to reduce CH_4_ emissions by 30% by 2030. Australian dairy systems could reduce CH_4_ emissions by 40–50% through various mitigation strategies. The accurate quantification of CH_4_ emissions from grazing cattle is essential for providing data for the national GHG inventory and developing effective mitigation strategies using rumen methanogenesis inhibitors. However, direct methane measurement using respiration chambers or invasive techniques such as the sulphur hexafluoride (SF_6_) method is challenging in grazing dairy herds. This study, performed in an Australian commercial dairy in Victoria, assessed the methane reduction potential of a polyphenol-rich sugarcane feed material (Polygain™) utilising an inverse-dispersion model in conjunction with an open-path laser technique. The findings of this study illustrate the potential of Polygain™ in reducing methane emissions from dairy cattle.

## 1. Introduction

Methane (CH_4_) is the second largest anthropogenic greenhouse gas (GHG) after carbon dioxide, with enteric CH_4_ from livestock, contributing to nearly 30% of global CH_4_ emissions [1,2]. The EU’s total CH_4_ emissions account for 4–5% of global CH_4_ emissions, and enteric fermentation from cattle accounted for approximately 70% of the agricultural CH_4_ emissions in 2020 [3]. In the U.S., dairy cows produced about 25% of the total enteric CH_4_ emission [4]. In China, enteric fermentation contributed over 40% of the total CH_4_ emissions in the agriculture sector [5]. In Australia, agriculture contributes roughly 13% of national GHG emissions, with the dairy sector—a key income-generating area—accounting for 12.5% of agricultural emissions, primarily from dairy cows [6]. Enteric CH_4_ comprises 57% of the emissions from the Australian dairy industry [7].

Continually increasing atmospheric CH_4_ emissions not only increases environmental pollution and accelerates global climate change but also negatively impacts animal productivity consequently leading to economic losses for farmers [8,9]. Over 100 countries have joined together through the Global Methane Pledge and committed to reducing CH_4_ emissions by 30% from 2020 to 2030 [10]. However, these mitigation efforts are hindered by the lack of accurate quantification of CH_4_ sources due to the difficulty in obtaining direct measurements [11]. Similarly, the Australian dairy sector aims to decrease GHG emissions intensity across the dairy industry by 30% by 2030 [12]. Therefore, reducing CH_4_ emissions from dairy cows represents a significant step toward meeting GHG reduction commitments [13]. Improvements in milk quality and quantity will assist farmers in increasing the value of milk sales [14].

One of the enteric CH_4_ mitigation approaches in ruminants is to develop a new emission reduction technique through the dietary feeding system while improving feed conversion efficiency [15,16]. The effectiveness of dietary substrates on CH_4_ emissions is mainly based on ruminal hydrogen concentration, the microbial population, fermentation, the rate of passage in the rumen, and the combination of these factors [17]. Previous studies reported that feeding lipid supplementation to pasture-based dairy cows can reduce CH_4_ emissions, such as whole oilseeds [18] and coconut oil [19]; however, the effects can vary with different lipid sources [4]. In addition, rumen modifiers/additives aimed at CH_4_ reduction may lead to undesirable outcomes, such as changes in microbial groups, or decreasing feed efficiency and profitability [5]. Plant-based supplements, known for their ability to modify the microbiota and reduce CH_4_ production, have shown effectiveness in ruminants [19,20]. These additives benefit the environment and offer economic advantages, including improved feed efficiency and enhanced growth rates.

Feed interventions and additives are increasingly used in ruminants to enhance productivity and reduce GHG emissions [21]. Rumen modifiers such as lipids, plant secondary compounds, and essential oils are used as feed supplements. Polyphenols are plant secondary metabolites synthesised through shikimate, acetate, and malvalonate/deoxy xylulose pathways [22]. Polyphenols affect rumen fermentation and methanogenesis, biohydrogenation, and milk yield and composition and possess an anti-inflammatory action [23]. Recent developments in our understanding of rumen methanogenesis have led to the repurposing of polyphenol plant extracts as feed additives for methane mitigation, but their primary usage as feed supplements in ruminant nutrition has been to increase animal production [24,25,26]. Methanogenesis inhibitors work by either directly preventing methanogenesis or by altering the rumen environment resulting in decreased methane production [27,28]. Specifically, polyphenols act on the ruminal microbiome by altering their cell membrane permeability, signal transduction and gene expression, thereby inhibiting enzyme synthesis and microbial colonisation [29,30,31]. This will lead to a leak of metabolites and ions that eventually results in leaking cells and microbe deaths [32]. In addition, polyphenols inhibit ruminal protein and fibre degradation and FA biohydrogenation [33].

There are different polyphenol plant extracts used as feed additives in ruminants for promoting productivity, enhancing performance and reducing emissions [34]. These compounds act by inhibiting the methanogen population and hydrogen-producing fibrolytic microbes [35,36]. Polygain™ (The Product Makers Australia) is a polyphenol-rich sugarcane feed material (PRSFM) being used in animal production systems to increase productivity and has shown promising results in reducing CH_4_ emissions in vitro, in sheep and dairy cows [37,38]. Besides the effect on CH_4_ emissions, Polygain™ is expected to improve meat and milk quality [39]. The antioxidants in Polygain™ may promote stable mitochondrial function, effective antioxidant properties and energy use to improve protein metabolism without affecting animal health [39,40,41]. Our previous study, which was a 1-month dose optimisation sheep study, including two different dosages of 1 or 0.25% Polygain™, showed enteric CH_4_ reductions of 49 and 33%, respectively [40]. Furthermore, the rumen microbiome analysis from this study (sequencing of the V3-V4 region of 16S rRNA) revealed a decrease in methanogen communities in the Polygain™-fed sheep.

Accurate and repeatable CH_4_ emissions measurement techniques are essential to characterise the current emissions for national GHG inventory based on prediction models and to evaluate and identify effective mitigation strategies. Many direct or indirect methods have been reported to quantify emissions from grazing animals [41]. The early measurement approaches used respiration chamber methods for individual animals and SF_6_ tracer-ratio techniques for a group of animals [42], but these methods have the disadvantages of being labour-intensive and restricting animal behaviour. On the other hand, micrometeorological methods have been developed for large grazing areas, for example, using the mass-balance method and nitrous oxide (N_2_O) tracer method with open-path Fourier-transform infrared spectroscopy (OP-FTIR) techniques to measure CH_4_ emissions from grazing cattle [43] and flux-gradient methods to measure emissions from grazing sheep [44]. Each of these techniques has advantages, disadvantages, and limitations, and their details can be found in many publications [45,46,47]. In an intensive rotational grazing system, a key challenge is relocating the measurement equipment daily as grazing animals between paddocks [48].

One of the options to calculate CH_4_ emissions in grazing cattle is the use of inverse-dispersion model (IDM). The IDM technique is a micrometeorological method in which emissions are calculated from gas measurements upwind and downwind of the animal paddock given the atmospheric wind and turbulent information. The principle is that CH_4_ emitted from the animals increases the downwind concentration levels above the upwind values, and emission rates are proportional to the downwind–upwind difference [49,50]. Compared with other methods to measure animal emissions (e.g., SF_6_ tracer-ratio method, respiration chambers, Greenfeed), IDM does not interfere with the animals or farm management practices, and it allows continuous monitoring [50]. Previous studies have shown that IDM combined with open-path laser concentration sensors can quantify CH_4_ emissions from groups of cattle [51]. This study aimed to quantify the CH_4_ emissions from dairy cows with and without Polygain™ supplementation using the IDM technique.

## 2. Materials and Methods

### 2.1. Animals and Experimental Design

The experiment was conducted at the University of Melbourne, Dookie Campus Dairy Farm (−36°19′60.00″ S, 145°41′59.99″ E), ~220 km north of Melbourne, Australia, between 25 November 2023 and 1 January 2024. The study was approved by the University of Melbourne Animal Ethics Committee (AEC ID: 2023-27376-46031-3). Thirty Holstein Friesian dairy cows in their 1–3 lactation (2–5 years old with an average body weight of 663.44 ± 6.07 kg and an average daily milk production of 28.99 ± 0.32 kg) were recruited for the feeding trial and data collection. The 30 cows were divided into two dietary treatment groups of 15 cows each: control (standard pellets) vs. treatment (pellets formulated by adding Polyphenol-Rich Sugarcane Feed Material to control pellets). Polygain™ is rich in polyphenols, amino acids, essential minerals and nutrients. It contains radicals such as peroxy radicals (33,000), hydroxy radicals (162,400), superoxide ion (45,100), singlet O_2_ (27,700) and peroxynitrite (7300), measured using the oxygen radical absorbance capacity (ORAC) assay, and expressed in µmol TE/100 g (TE as Trolox equivalents). The mineral composition (mg per 100 g) is potassium—2000–4000, sodium—50–200, calcium—300–500, magnesium—3000–5000, iron—10–15, chromium—0.2–0.5, selenium—0.05, and zinc—0.5–1.5. The energy, protein, total fat, and carbohydrate concentration per 600 kJ is 100 g, 6.9 g, <0.1 g, and 26.6 g, respectively [52]. Polygain™ exerts its action through interaction with gut microbiota [53]. The control pellets were formulated as per the standard formulation (~16% crude protein), and treatment pellets were formulated with the addition of Polygain™ (0.25% of the pellet) to the control pellets. Experimental diets were fed for 5 weeks, including 3 weeks of the adaptation period. After the feeding trial, all cows resumed their normal feeding and farm practices and were monitored for one additional week. Other management practices during the study period were the same for all cows as normal farm practices. More information about feed intake and milk production is shown in Table 1. The composition of the concentrate and rye-grass pasture forage are shown in Table 2 and Table 3. The dry matter requirement was estimated at 3% of body weight [54].

The terrain around the dairy farm was flat, with bare soils or short grass. The mean daily minimum/maximum air temperature was 9.4 and 20.9 °C, respectively, and the annual rainfall was 548.7 mm [55]. Milking was performed using robotic milking machines. The milking shed and feeding systems have a capacity for 180 cows. Animals were grazed in the irrigated rye-grass pasture (43 hectares). Animals’ waste during milking periods was collected every day, and the liquid fraction of the waste was immediately separated from the solids through a facility next to the milking shed. The liquids were stored in the tank for irrigation or washing the milking shed ground, while the solids were taken away and used for bedding materials in the calves’ paddocks. The measurement site was located ~500 m south of the milking shed; other cows only grazed north of the site during the measurement period, and there were no other CH_4_ sources within 100 m upwind of the experimental site.

The first three weeks were the animals’ adaptation phase (25 November–15 December 2023), followed by the two weeks’ CH_4_ emission measurement phase (16 December 2023–1 January 2024). Throughout the study, the animals grazed in their regular production environment and were milked in the milking shed twice a day. Polygain™ incorporated in the standard pellets was fed to treatment cows, while control cows were fed standard pellets. Body weight was recorded daily, including before the administration of Polygain™ supplementation. After 5 weeks, the cows were released back into the Dookie commercial herd and monitored for one more week to observe their normal behaviours.

**Figure 1:**
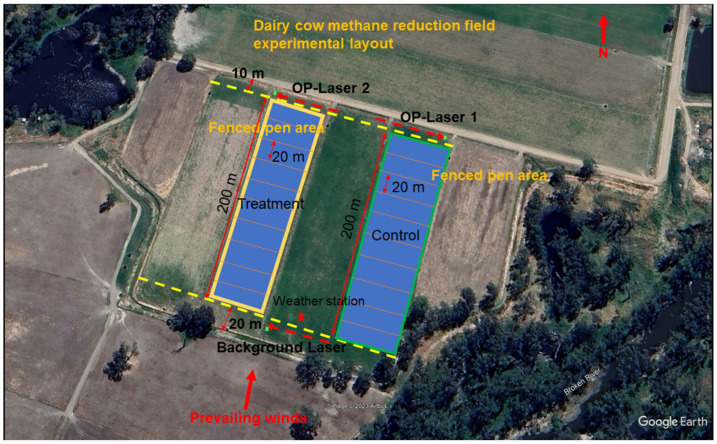
The layout of the experimental site at Dookie Campus Dairy Farm. Each day, two grazing areas (20 × 50 m) were created for the treatment and control paddock, with a temporary fence and water trough (upper panel). CH_4_ measurements were only taken during southerly winds. The locations of the weather station, upwind and downwind lasers, and retro retroflectors are also shown. (source: Google map).

The experimental grazing paddocks were constructed within four large paddocks throughout the measurements. Before the measurement, these paddocks were irrigated. Two paddocks were used for the first 6 days of measurements, for both control and treatment groups. Another paddock was used for the rest of the 8 days of measurements. Shifting the paddocks was to avoid the tree lines located to the S and SE of paddocks so that the surroundings and atmospheric conditions remained similar (Figure 1). On each measurement day, cows grazed at two parallel fenced sub-paddocks of 20 m by 50 m, separated by 60 m, except afternoon milking time, then moved to the next sub-paddock to access fresh pasture in the following day after morning milking time. The time when cows left and returned to the paddocks was recorded daily. There was a total of 14 pairs of sub-paddocks during the emission measurement period. This parallel set-up allowed not only the comparison of the emissions from the two groups at the same geometrical and wind conditions but also minimised the cross-contamination of CH_4_ emissions between the two groups. The rotation of grazing paddocks also required the measurement equipment to be shifted to the next sub-paddock.

### 2.2. Methodologies

#### 2.2.1. Inverse-Dispersion Method (IDM)

We used IDM to measure CH_4_ emissions from the dairy grazing cows [56]. An atmospheric dispersion model (software WindTrax 2.0, Thunderbeach Scientific, Concord, ON L4K1K8, Canada) used the IDM method based on the Monin–Obukhov similarity theory (MOST) to calculate the relationship between the flux (*Q*) from the source area and the enhanced CH_4_ concentration (Δ*C*) between downwind and upwind of the source [57]. The calculation followed the equation below (Equation (1)):

*Q* = (*C*_downwind_ − *C*_upwind_)/(*C*/*Q*) _sim_ (1)

where Q is the CH_4_ emission rate (μg/m^2^/s), *C*_downwind_ and *C*_upwind_ are CH_4_ concentrations measured downwind and upwind of the grazing area, respectively, and Δ*C* = *C*_downwind_ − *C_upwind_*, (C/Q) _sim_ is the ratio of concentration and emission rate.

The input information for the WindTrax model included line-averaged concentrations upwind and downwind of the source area, wind turbulence data, and the coordinates of the source area (sub-paddocks) and all the sensors for the measurements.

#### 2.2.2. Instrumentations

A tunable diode laser absorption spectroscopy procedure was used to distribute three open-path laser sensors. The line-average CH_4_ concentrations are measured along the open measurement path between the laser transmitter and the retro-reflector using a transmitter and a 6-corner cube retro reflector. This technique has been used to measure CH_4_ concentrations in cattle in the past [57,58,59]. Two CH_4_ concentration sensors (OPL33 and OPL34, Unisearch Ltd. Pty, Canada) were placed 10 m north of the grazing paddocks, each with a path length of 60 m (one way). The third concentration sensor (OPL1013, Gasfinder 2.0, Boreal Laser, AB, Canada) was positioned upwind of the source areas, 20 m north of the farm fence line, with a path length of 55 m (one way) (Figure 1). The detection limit for the sensors is < 0.3 and 10 ppb at 100 m for the Unisearch laser and the Boreal laser, respectively, with a response time of 1–2 s. Unisearch open-path lasers have been reported with better precision and performance compared with other sensors (>0.01%) [60]. Additionally, the sensor also has a ±2% signal detection limit, and the accuracy is ± 5% based on the information from the laser manufacturer.

At the south of the paddocks, the weather station was set up. A three-dimensional (3D) sonic anemometer (CSAT3, Campbell Scientific, Logan, UT, USA) was also set up at 2.61 m height above the ground. Fifteen-minute average climate data including ambient temperature, ambient pressure, and wind statistics, such as wind speed, wind direction, wind variation, and covarions, were recorded by a datalogger (CR23X, Campbell Scientific, Logan, UT, USA) at a frequency of 10 Hz. Atmospheric stability parameters of friction velocity (u_*_), surface roughness (z0), and Obukhov stability length (L), and the standard deviation of wind speed component u, v, and w divided by u_*_, σu/u_*_, σv/u_*_, and σw/u_*_, respectively, were retrieved from these sonic data.

#### 2.2.3. Cross-Calibration of Concentration Sensors

Three CH_4_ concentration sensors were set up next to an animal paddock and run side-by-side for two days before the flux measurements aiming to calibrate the difference between these sensors. During the cross-calibration process, we chose OPL34 as the reference sensor and a linear correlation was applied to examine the data between OPL33 and OPL34, and between OPL1013 and OPL34. Each slope of the correlation was obtained as their correction factor. We then forced the measurements of OPL33 and OPL1013 to be the same as/close to OPL34 by multiplying the correction factor. The corrected line-averaged concentrations and wind statistics were processed over 15-minute intervals using the software SAS (SAS 9.4, SAS Institute Inc., Cary, NC, USA). The laser sensors were not calibrated against the external references as the emission rate calculations in this study were associated with the absolute change (Δ*C*) in concentrations.

In our study, we did not calibrate the IDM. The combination of IDM with open-path spectroscopic-based concentration sensors is recognised as a powerful approach to measuring emissions, and the precision of the IDM method was sufficient for detecting emission differences from different cattle diets [61,62]. Numerous researchers have reported their studies on comparing IDM (Windtrax) with the “standard” tracer method, e.g., using CH_4_, SF_6_, or N_2_O as the trace gas with a known release rate to quantify CH_4_ emissions from grazing cattle and approved that the agreement between the measurements was within 10% [60,63].

#### 2.2.4. Data Filtering

The 15-minute averaged data (concentrations and wind turbulence) were filtered when poor-quality periods corresponded to atmospheric boundary conditions that do not satisfy the assumptions of the MOST in the dispersion mode. The horizontal homogenous surface layer could be determined by the statistical wind properties, such as some key surface observations, u_*_, L, z0, and wind direction. It has been approved that screening these data can improve the accuracy of IDM estimates by using the filtering threshold of u_*_ < 0.15 m/s, |L| < 10 m, and z0 > 0.9 m to identify these invalid conditions (rapid atmospheric change and stable stratification), but consequently, fewer data points were retained [63,64]. In this study, we applied the filtering criteria: the IDM trajectories touchdown in the source area ≥ 20%, touchdown in another area < 5%, u_*_ > 0.15 m/s, |L|> 2 m, z0 < 0.9 m. To avoid contamination from other sources, the measurements during northerly winds were not counted for the flux calculation. In addition, the tree lines at S and SE of the experimental paddocks could disturb the upwind air flow, the suitable wind direction, therefore, was chosen at a range of between 110° and 250°. Further, good-quality concentration data were used for flux calculation when the laser signals (light level) were strong, >15% of the power for Unisearch lasers or between 3000 and 15,000, with a strong linear relationship between the collected spectrum and the built-in reference cell, R^2^ > 96 (Boreal Laser).

#### 2.2.5. Statistical Analysis

The effect of Polygain™ supplementation on methane emission was analysed using a linear mixed-effect model with treatment (Polygain™ vs. control) as the fixed effect and days of measurement as a random effect using the lmer function in lme4 package in R (Version 4.4.2) [64]. The results were plotted using ggplot2 and ggrepel packages. The significance level was set at *p* < 0.05. The model assumptions were checked using the diagnostic residual plot. Residuals were randomly scattered in the residual plot with no apparent patterns.

## 3. Results

### 3.1. Milk Production, Animal Weight Gain, and Feed Intake

During the gas measurement phase, the animals’ milk production and body weight from the treatment and control groups are given in Table 4. Daily milk production, body weight and total intake did not significantly vary between the treatment and control group.

### 3.2. Climate Data

**Figure 2:**
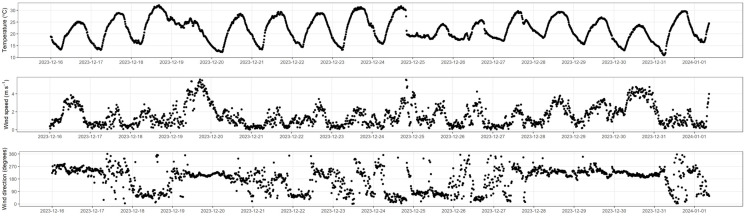
Wind speed, wind direction, and ambient temperature measured at the Dookie farm from 16 December 2023 to 1 January 2024.

During the measurement period (16 December 2023 to 1 January 2024), the 15-minute wind speed, wind direction, and ambient temperature are shown in Figure 2. During the measurement period, the mean wind speed was 1.54 m s^−1^ (number of observations, n = 1572), and the mean ambient temperature was 21.8 °C (n = 1572); about 57% of measurement was obtained during southern winds.

### 3.3. Fifteen-Minute Average CH_4_ Concentrations

**Figure 3:**
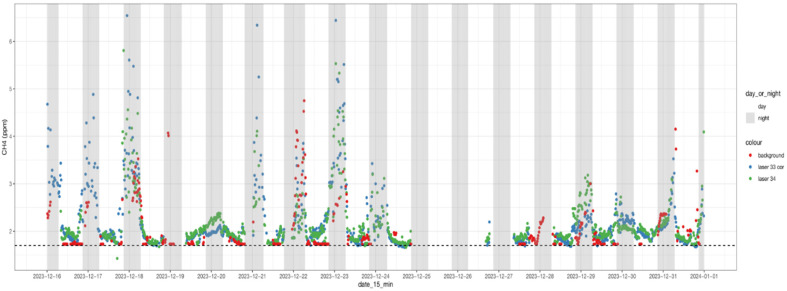
Fifteen-minute averaged CH_4_ concentrations of control cows (green), treatment cows (blue), and background levels (red) over the measurement period from 16 December 2023 to 1 January 2024. The dotted line represents 1.7 ppm.

Filtered 15 min CH_4_ concentrations measured from the treatment and control group are shown in Figure 3. The dataset showed a discontinued time series of concentration measurements mainly due to the storms, the period we had to turn off the equipment, and unfavourable wind directions.

### 3.4. CH_4_ Fluxes and Total GHG Emissions (CO_2_ Equivalents, CO_2_-e)

**Figure 4:**
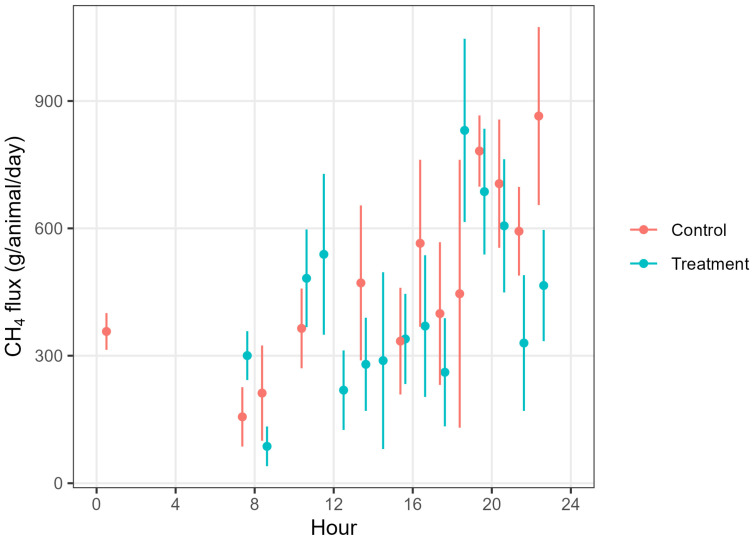
Hourly variation in methane production (g/day/animal) in control and treatment cows during the experimental period.

**Figure 5:**
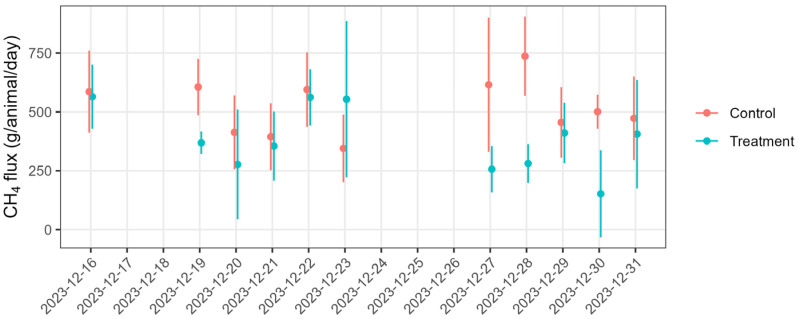
Daily average CH_4_ emission rates from the control group (blue) and treatment group (red) were measured by the inverse-dispersion model (IDM) coupled with open-path lasers from 16 December 2023 to 1 January 2024. The error bars represent the standard deviation of the mean.

The measured concentrations were filtered following the filtering criteria, and the useful data were used to calculate 15-minute interval CH_4_ emission rates. There were 116 and 94 good datasets counted for the flux calculation, accounting for 26 and 21% of the useful data for the treatment and control groups, respectively. This was sufficient to represent the emissions from the source area. The data gap due to the filtering or instrument downtime was filled to minimise the potential bias in the flux calculations caused by irregular emission sampling combined with the diurnal pattern of cows’ emissions. We found that hourly CH_4_ emissions from both treatment and control cows showed a diurnal variation (Figure 4): higher emissions in the evening and low or no detectable emissions at night. However, there were no obvious correlations between the fluxes and wind friction velocity (u*). The 15-minute emission data were grouped into 3-hour time bins over the day, and daily emissions were calculated from the sum of the bin averages. Daily CH_4_ fluxes from both control and treatment cows varied over the measurement time (Figure 5). We calculated the mean daily CH_4_ flux of 494.9 ± 12 (mean ± standard error, SE) (n = 94) and 376.6 ± 12 (n = 116) g CH_4_/animal/day for the control and treatment cows, respectively, over the 16-day measurement period.

**Figure 6:**
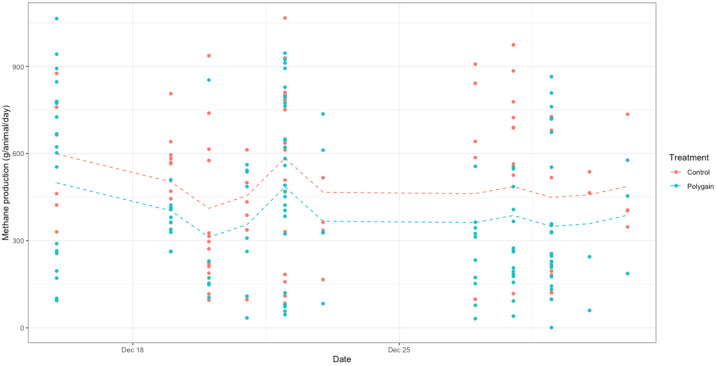
The linear mixed-effect model shows a significant effect of Polygain™ supplementation in dairy cows (*p* < 0.05). Treatment (control vs. Polygain™ (n = 15 cows in each group) is shown as the fixed effect and the measurement days are shown as a random effect.

There was significant variation in methane production between the control and treatment groups (*p* < 0.05), as shown in Figure 6. In the linear mixed-effect model, methane production (MP, g CH_4_/animal/day) was 494.9 ± 12 and 376.6 ± 12 for the control and treatment cows, respectively. We estimated the DMI, based on body weight, to be 19.88 and 19.93 kg for the treatment and control group, respectively. The methane yield (MY—methane emission per kg DMI expressed as g CH_4_/animal/kg DMI) was 18.96 for the treatment group and 24.84 g for the control group. Furthermore, we calculated methane intensity (MI, g CH_4_/animal/kg milk) to be 13.01 ± 0.4 and 17.04 ± 0.4 for the treatment and control groups, respectively. The CH_4_ fluxes expressed as various methane phenotypes from both treatment and control cows are summarised in Table 4. The effect of Polygain™ feed additive on CH_4_ reduction was observed in our study; it reduced MP by 24% and MI by 24%.

## 4. Discussion

This study showed that Polygain™ supplementation significantly reduced methane emissions in dairy cows. The methane production values obtained in the present study were higher than the emissions reported in the IPCC emission factor (a default value of 100 kg/animal/year^−^ for Oceania countries, 128 kg/animal/year for North America), and other studies [65,66,67,68,69], but comparable to the higher range of the emissions reported [70]. Different genetic groups, such as purebred vs. crossbred, dry cows vs. lactating cows, and dry cows and late lactating cows, produce varying enteric emissions [71,72,73]. The methane yield obtained in the study was comparable to the low range of 18.2 g CH_4_/animal/kg DMI in New Zealand [74] and the higher range of 23.3 g CH_4_/animal/kg DMI in Brazil [73].

We observed that downwind concentrations from both groups varied over time, at a range of 1.76 to 6 ppm, and followed a similar diurnal pattern: higher concentrations at night-time and lower concentrations in daytime, which is associated with atmospheric turbulent conditions and animal activities [74,75]. The similar temporal variations between the two groups also reflected their similar activities. The background concentrations of the experimental site fluctuated over time, with an average CH_4_ concentration of 1.73 ppm (Figure 3). The enhanced CH_4_ concentrations between the treatment and control group above the background level varied from 5 to 900 ppb [76,77].

The temporal variation in daily emissions in the present study could be attributed to animal activities, such as grazing or resting [78], the source locations and heights could be varied [79], and unsteady wind direction and wind speed over the averaging period can contribute to the variation [80]. The temporal variation in emissions can be attributed to the changes in environmental conditions, including wind direction, wind speed, and animal activities, such as drinking, eating, and resting. In a previous study, unsteady wind direction and wind speed over the 15-minute averaging period contributed to the variability of the emissions [80]. A previous study demonstrated a 24-hour diurnal variation in cattle emissions, and higher emissions occurred about 2 h after feeding time and lower emissions at night-time when animals rested most of the time, especially under stable atmospheric conditions [78,81]. The other factors are the height of the concentration measurement and the source locations [82,83,84].

Because polyphenols are abundant in plant secondary metabolites like tannins, saponins, alkaloids, and essential oils, they have a long-term effect on animal health, production, and microbial diversity [23,34]. These effects were found to be dose-dependent [85]. Polyphenol-rich plant extracts such as Polygain™ can reduce enteric methane emission by 8–50%; however, factors, such as palatability and method of extraction, need further investigation [86]. The methane emission reduction rate in response to Polygain™ supplementation in our study was lower than that fed with other plant-based feed additives, such as oregano, tannins, and seaweed (>50%) [25,85], but higher than the range of 4–7% of reductions using the additives, including dietary medium-chain lipids [86,87,88,89] and other plant secondary metabolites [90,91]. A blend of cinnamaldehyde, eugenol, and capsicum oleoresin reduced CH_4_ emission by only 3.4% [92]. However, many of the studies with emission reduction showed variable reduction due to the inconsistent source supply or the composition of the diet [93]. The additives with methane reduction showed dose-dependent efficacy and higher doses led to effects on rumen physiology such as enhanced rumen H_2_ production [94].

The reduction of CH_4_ emission per cattle was calculated at 118 g per day. Using a global warming potential value of 28 for CH_4_, we estimated a total GHG emission rate, equivalent to CO_2_ (CO_2_-e) of 1.21 tonnes per annum per cow, with an average body weight of 645 kg. Based on our measurement, we extrapolated our emission reduction to the national dairy industry. With a national dairy cattle population of 1.44 million [95], we estimated that the reduction of CH_4_ emissions from the Polygain™ fed dairy industry across over 5000 dairy farms (1.34 Mt CO_2_-e) contributes 2.62% reduction in Australian agricultural emissions (66.4 Mt CO_2_-e) [96]. This is a further step moving forward to our emission reduction target [97,98,99].

The reduction in CH_4_ emissions in dairy cattle following Polygain™ supplementation in this study further confirmed its anti-methanogenic efficacy across species, which we have reported before in our previous sheep study [38]. Polygain™ can mitigate enteric CH_4_ production through several mechanisms, including the inhibition of methanogen activity and altering the rumen’s fermentation process. This can be seen from the CH_4_ fluxes per milk production that was also reduced from the treatment group compared to the control group [100].

Despite the growing promotion of feed additives, such as chemical anti-methanogens, to reduce methane emissions in ruminants, their widespread use may depend on their efficacy, safety, and effects on the economy and environment [101]. Bromoform and 3-nitroxypropanol (3-NOP) are examples of chemical methane inhibitors that work by preventing methanogenic archaea from growing in the rumen [25]. A promising approach that calls for more thorough research is the investigation of the synergistic effect of different plant extracts such as Polygain™, with other inhibitors to expand the usage of methane-inhibiting feed additives in ruminants.

## 5. Conclusions

In the present study, we found that Polygain™ supplementation significantly reduced CH_4_ emission in commercial dairy cows by 24%. Hence, Polygain™ can be integrated into regular feeding routines, typically added to the feed in specific proportions based on the type and age of the livestock. Thus, the use of feed additives like Polygain™ represents a promising approach to addressing climate change by targeting one of the major sources of agricultural GHG emissions. The IDM method is a useful tool for quantifying CH_4_ emissions simultaneously from two herds of dairy cows with no interference with animals. Furthermore, the IDM method is particularly valuable in quantifying a whole herd outcome of feeding a methane-inhibiting supplement, as the result invariably integrates individual animal behaviour and variation in individual intake. The dairy sector is campaigning for lower CH_4_ emissions from farming systems to satisfy GHG emission reduction targets. Future studies with longer supplementation and measurements are required to investigate the long-term efficacy and sustainability of Polygain™ and assess its performance under varying climatic and management conditions. Additionally, a cost-benefit analysis is needed to gain insights into the economic aspects, for wider use of this mitigation strategy at various production systems.

## Figures and Tables

**Table 1:** Milk production, live weight, and feed allocation for control and treatment cows.

Parameter	Control (Mean ± SD)	Treatment (Mean ± SD)
Lactation days	130.2 ± 81.8	121.3 ± 84.6
Lactation no	3.9 ± 1.1	4.1 ± 2.1
Body weight (kg)	656.61 ± 7.7	656.90 ± 5.5
Day production (kg)	29.45 ± 0.3	29.48 ± 0.4
Total concentrate given (kg DM)	10.8 ± 0.6	10.8 ± 0.8
Daily concentrate remaining avg (kg)	1.4 ± 0.7	1.5 ± 0.7
Daily concentrate consumed avg (kg)	9.4 ± 0.7	9.4 ± 0.8

**Table 2:** Composition of the concentrate fed.

Elements/Nutrients/Vitamins	As Fed
Crude protein	14.25%
Crude fibre	12.0%
Crude fat	1.5%
Calcium	1.0%
Phosphorus	0.5%
Magnesium	0.3%
Salt	0.5%
Copper	45 mg/kg
Zinc	150 mg/kg
Selenium	0.5 mg/kg
Manganese	70 mg/kg
Iodine	1.0 mg/kg
Cobalt	1.5 mg/kg
Vitamin A	6500 iu/kg
Vitamin D3	800 iu/kg
Vitamin E	15 mg/kg

**Table 3:** Composition of the rye-grass pasture forage fed.

Elements/Nutrients/Vitamins	Composition
Dry matter (DM)	93.5%
Moisture	6.5%
Crude protein	18.3%
Acid detergent fibre	25.7%
Neutral detergent fibre	48.1%
Digestibility (DMD) **^#^**	71.3%
Digestibility (DOMD-calculated)	67.2%
Metabolizable energy (calculated)	10.6 MJ/kg DM
Water soluble carbohydrates	6.8% of DM
Fat	5.2% of DM
Ash	13.2% of DM

^#^ DM, dry matter; DMD, dry matter digestibility; DOMD, digestibility of organic matter in the dry matter.

**Table 4:** Daily milk production (DMP) and body weight (BW) are shown during the gas measurement from 16 December 2023 to 1 January 2024. CH_4_ fluxes in methane production (MP, g CH_4_/animal/day) and in methane intensity (MI, g CH_4_/animal/kg milk/day) are also shown.

Period	DMP (kg)		BW (kg)	
	Treatment	Control	Treatment	Control
week 4	29.45	29.48	656.90	656.61
Week 5	28.70	28.78	662.66	664.52
Week 6	28.68	28.85	667.95	671.97
Average	28.94 (±0.3) ^&^	29.04 (±0.2)	662.50 (±3.2)	664.37 (±4.4)
	**MP** g CH_4_/animal/day		**MI** (g CH_4_/animal/kg milk/day)	
	**Treatment**	**Control**	**Treatment**	**Control**
	376.6 (±12) ^&^	494.9 (±12) ^&^	13.01 (±0.4)	17.04 (±0.4)

DMP, daily milk production (kg); BW, body weight (kg); MP, mean daily CH_4_ production (g CH_4_/animal/day); MI, mean daily CH_4_ intensity (g CH_4_/animal/kg milk/day). Treatment group: 15 cows with Polygain™ in feed; control: 15 cows without Polygain™ in feed. ^&^ mean (± standard error).

## Data Availability

Data are available upon request to the corresponding author.

## Abbreviations

The following abbreviations are used in this manuscript:

GHG	Greenhouse gas emissions
CH4	Methane
N2O	Nitrous oxide
PRSFM	Polyphenol-rich sugarcane feed material
OP-FTIR	Open-path Fourier-transform infrared spectroscopy
IDM	Inverse-dispersion model
OPL	Open-path laser
MP	Methane production
MI	Methane intensity
DMP	Daily milk production
BW	Body weight
CO2-e	CO2 equivalents
